# The Voice of Patients Really Matters: Using Patient-Reported Outcomes and Experiences Measures to Assess Effectiveness of Home-Based Integrated Care—A Scoping Review of Practice

**DOI:** 10.3390/healthcare11010098

**Published:** 2022-12-28

**Authors:** Ewa Bandurska

**Affiliations:** Center for Competence Development, Integrated Care and e-Health, Medical University of Gdańsk, Debowa 30, 80-208 Gdansk, Poland; ebandurska@gumed.edu.pl

**Keywords:** chronic obstructive pulmonary disease (COPD), depression/mood disorder, managed care, multidisciplinary care, obesity, patient adherence

## Abstract

Background: The aim of the study is to analyze the prevalence of using patients’ reported outcomes measures and experiences (PROMs and PREMs) in relation to integrated care (IC). Material and methods: To select eligible studies (<10 years, full-text), PubMed was used. The general subject of the articles referring to the type of disease was indicated on the basis of a review of all full-text publications discussing the effectiveness of IC (N = 6518). The final search included MeSH headings related to outcomes measures and IC. Full-text screening resulted in including 73 articles (23 on COPD, 40 on diabetes/obesity and 10 on depression) with 93.391 participants. Results: Analysis indicated that authors used multiple outcome measures, with 54.8% of studies including at least one patient reported. PROMs were more often used than PREMs. Specific (disease or condition/dimension) outcome measures were reported more often than general, especially those dedicated to self-assessment of health in COPD and depression. PROMs and PREMs were most commonly used in studies from the USA and Netherlands. Conclusion: Using PROMS/PREMS is becoming more popular, although it is varied, both due to the place of research and type of disease.

## 1. Introduction

Analysis of the effectiveness of care has traditionally focused on objective clinical indicators. According to international HTA (health technology assessment) guidelines, to conduct research on the effectiveness of treatment, hard endpoints, followed by clinically relevant surrogate points, must be used [1]. Today, the discussion on the role of other outcomes is open and ongoing. Using Patient-reported outcome measures (PROMs) and patient-reported experience measures (PREMs) seems to be increasingly relevant for assuring a good quality of care and supporting gathering knowledge considering disease course. It is believed that their role is particularly important in patient-focused care models, i.e., in integrated care and integrating treatment methods from various areas [2,3]. Integrated care is currently accepted as a form of care around the world; however, its understanding and definition may vary depending on the perspective or purpose for which such a definition is built [4]. A health system-based definition provides that it should be understood as: “health services that are managed and delivered so that people receive a continuum of health promotion, disease prevention, diagnosis, treatment, disease-management, rehabilitation and palliative care services, coordinated across the different levels and sites of care within and beyond the health sector, and according to their needs throughout the life course” [5]. Integrated care is provided through integrated care programs that proactively organize and coordinate the comprehensive delivery of both health and social care services, aiming to improve patient outcomes and reduce healthcare expenses [6].

PROMs are questionnaires or scales that allow for the measurement of the results of treatment from the patient’s perspective [7]. They can be divided into generic, disease-specific and condition/dimension-specific [8,9,10]. PREMs bring information on how patients conceive medical care. Relational PREMs deal with relations between patients and medical personnel, patients’ expectations and preferences. Functional PREMs are related to basic expectations about technical issues related to delivering healthcare [11]. Information obtained from tools such as PROMs and PREMs are used in many ways: in scientific research, projects improve the quality of care and audits and are conducted for pharmacoeconomic purposes [12]. They allow the management of healthcare in a patient-centric manner and gain feedback from healthcare providers that can be used in building quality improvement strategies [13]. Moreover, they allow clinicians to better understand patients and identify those health outcomes that are crucial from the patient’s point of view [14]. The collection of this type of data is supported by researchers who indicate that attachment to routinely used outcome measures can lead to a marginalization of patient needs and implementation of treatment that will be characterized by low compliance [15,16]. Although the importance of patient-centered outcomes is unquestionable, some doubts are raised considering the reliability of data. The quality of measurement is determined by how data is obtained and with the use of research tools [17]. It was found that there are statistically significant differences in assessing the relevance of symptoms by a doctor and a patient [16,18]. Information collected through PROMs and PREMs can be used in many areas: providing individual medical care of good quality or supporting the decision-making process in managing healthcare. 

The relevance of this study for understanding the idea and role of patient-centric care is significant as it provides a comprehensive set of basic knowledge about research using data coming directly from patients, identifies areas in which their role is already proven, and where there is still a need to work on increasing the dissemination of usage of such indicators. Moreover, the collection of data based on patient-reported measures might result in numerous positive results, among others: support for communication between medical staff and patients, increase in patient satisfaction and compliance, and enabling patients to control their own health condition better. These elements are crucial for providing effective integrated care and, in a broader aspect, a successful healthcare system based on a value-based approach. 

## 2. Material and Methods

The main research question in this scoping review was what is the prevalence of using outcomes measures reported by patients remaining under the support of integrated care (IC). The paper also sets specific questions:

1. What types of PROMs and PREMs are used to describe the effectiveness of IC programs? Are there differences in the use of PROMs and PREMs depending on the type of disease and the country?

The studies included in the analysis concerned:

2. The patients suffering from one of three chronic diseases: COPD, obesity/diabetes or depression, who received integrated care.
3. The intervention discussed was integrated care, which was assessed by researchers in terms of its effectiveness.

The comparison included an analysis of type, time and place of outcomes measures reported by patients receiving integrated care. Additionally, all indicators that were used by researchers to describe the IC used were subject to a preliminary quantitative assessment. Outcomes measures were a basic element of the analysis, along with an assessment of the frequency of using PROMs and PREMs, their type, the place of conducting the research and the date of publishing.

**Figure 1:**
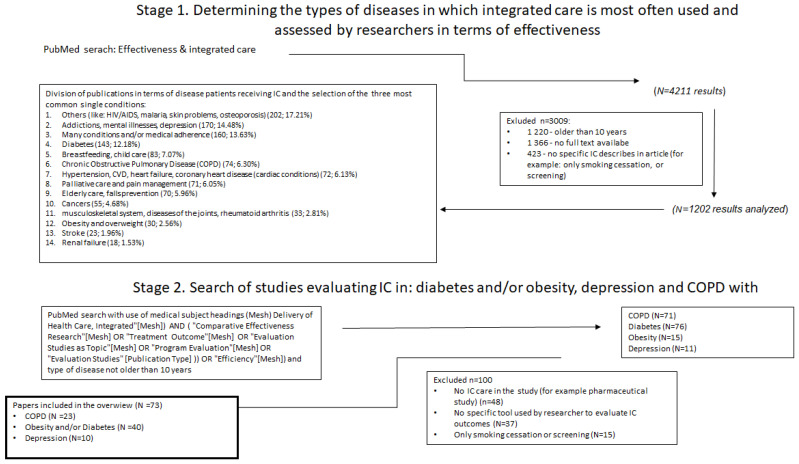
Search strategy.

The study design included two stages (Figure 1). 

Due to the fact that many PROMs and PREMs are dedicated to a specific disease, PubMed first searched to determine which chronic conditions research on the effectiveness of integrated care is carried out most often. Analysis of 6518 available full-text articles indicated that in the first place, most often, these are mental diseases and addiction to psychoactive substances 14.48% (mostly depression), many coexisting conditions (13.63%), diabetes and/or obesity (12.18%), breastfeeding and childcare (7.07%), chronic obstructive pulmonary disease—COPD (6.30%), palliative care and chronic pain (6.05%) and others (including among others: malaria, HIV, bedsores, infectious diseases, osteoporosis, allergies, psoriasis, multiple sclerosis)—17.21%. As a result, the overview was performed on the three most common diseases—diabetes and/or obesity, depression and COPD. 

Stage two of the search included medical subject headings (“Delivery of Health Care, Integrated” [Mesh]) and selected chronic conditions: “Pulmonary Disease, Chronic Obstructive”, “Diabetes Mellitus”, “Obesity”, “Depression” limiting the search strategy to “Comparative Effectiveness Research” [Mesh], “Treatment Outcome” [Mesh] or “Program Evaluation” [Mesh]). The inclusion criteria were: articles not older than 10 years, full text available and English language. The exclusion criteria were all those that did not comply with the inclusion criteria, studies not using specific research tools to assess the effect or did not assess IC and studies evaluating pharmaceutical and/or surgical procedures. Protocols of currently planned studies were enrolled in the analysis. 

It resulted in identifying 173 articles. Full-text screening resulted in excluding 100 papers based on inclusion and exclusion criteria. Among the remaining 73 articles, 23 considered COPD, 40 diabetes or obesity and 10 depression. 

For each of the identified studies, general characteristics and data on elements used in IC were extracted. The analysis included only those articles in which authors indicated specified outcome measures for assessment of clinical end-point, PROMs and/or PREMs or utilization of healthcare resources. The frequency of using patient-reported outcomes measures and experiences (divided into the following groups: generic, disease-specific, condition/dimension-specific) was analyzed in relation to the country, year and type of disease.

## 3. Results

### 3.1. General Characteristics of the Studies

The search identified 73 studies from the years 2009–2019 that were eligible for the analysis. This search enrolled a total of over 93.391 thousand patients. The median number of patients within the study group was 263 (ranging from 20 to 17.142). Most of the studies were original studies on the evaluation of integrated care models, and the newest studies (mostly from the years 2017–2019) also studied protocols planned for realization. The eligible studies described models of IC offering various forms of support for patients, usually more than one (85%) (Table 1 and Table 2).

### 3.2. Outcomes Measures Used in Studies

The assessment of care was reported in all studies, but not all of them used PROMs or PREMs for assessing effectiveness (54.8% used at least one PROM or PREM). The most common approach was to analyze from 6 to 10 different outcomes.

### 3.3. PROMs and PREMs in Evaluation of IC

PROMs or PREMs were reported in 39 studies, in 24 of which outcomes were described with PROMs, in 11 both PROMs and PREMs and in 4 with exclusive use of PREMs. Specific (disease or condition/dimension) outcomes were reported more often than general (22 vs. 8). The most commonly used PROM was the Medical Research Council scale (mMRC) and St. George’s Respiratory Questionnaire (SGRQ) (both N = 8). For details see Table 3. 

**Figure 2:**
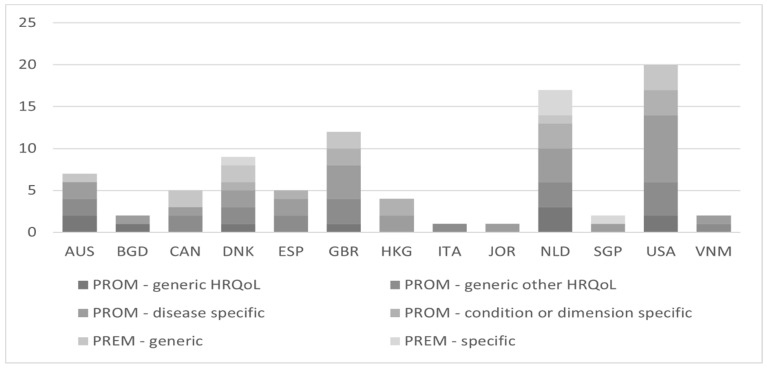
Number of PROMs and PREMs used in studies by country.

As shown in Figure 2, PROMs and PREMs were most commonly used in studies from the USA, followed by the Netherlands. In ten studies from the USA, twelve different tools were used to assess patients’ outcomes or experiences, out of which PHQ-9 was the most popular (N = 7). In six studies from the Netherlands, eight different tools were used—most often CCQ (N = 5). All types of patient-reported indicators were present in studies from Denmark and the Netherlands. 

**Figure 3:**
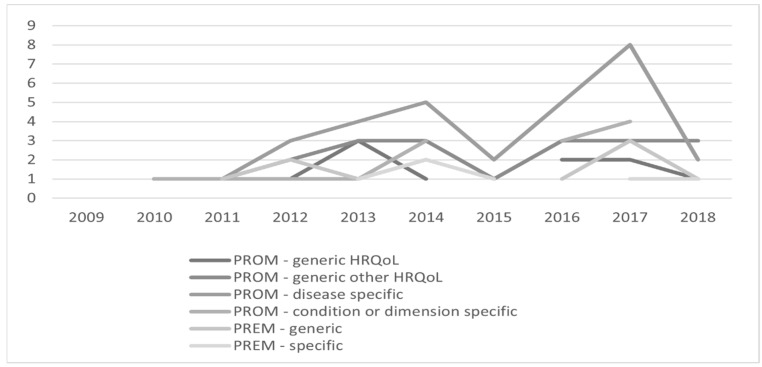
Number of PROMs and PREMS in studies by year of publication.

As shown in Figure 3, the first studies using specified questionnaires for assessing PROMs and PREMs were published within the analyzed period of time in 2010, and these two studies used only condition- or dimension-specific PROMs. The highest number of PROMs and PREMs was noted in 2017, mostly due to the widespread use of disease-specific PROMs.

**Figure 4:**
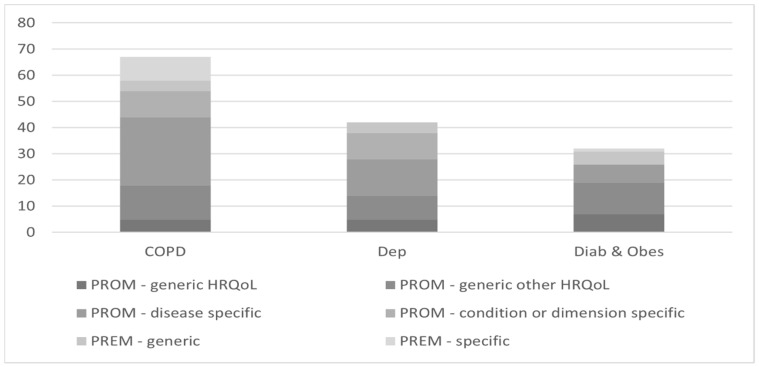
Number of PROMs and PREMS in studies by type of disease.

As shown in Figure 4, all types of PROMs and PREMs were reported as measures only in studies dealing with COPD. Likewise, PROMS were used most frequently in the assessment of IC dedicated to this disease (18 of 23 studies, 78.2%). Studies related to depression did not mention specific PREMs, and those related to diabetes did not use specific PREMs nor condition/dimension PROMs. 

## 4. Discussion

Reliable assessment of care, including integrated care, requires a multi-criterial approach. There is an increased interest in using the information provided by patients considering their health and experience related to healthcare [91,92]. Contemporary understanding of healthcare goes far beyond just providing health services. It is increasingly indicated that the health system must be designed to achieve health goals that are important to patients [91,93,94], which requires using PROMs and PREMs. The presented article can be considered a compendium of knowledge about the available indicators and the desired direction of further research aimed at the improvement of the effectiveness of integrated care. It identifies areas where research activities are particularly needed, which can be an inspiration for further research. It also allows you to familiarize yourself with a wide range of PROMs and PREMS questionnaires, indicating the added value of their use in healthcare management without overlooking them [12]. It is clear that various integrated care programs have been and are being evaluated in terms of clinical and economic effectiveness for many years, and the effectiveness of such interventions in these aspects has been repeatedly demonstrated [95,96,97]. However, the presented overview of practice in the assessment of IC indicates a different approach to this aspect, directing attention to the perspective and subjective feelings of patients, which resulted in some interesting observations. First of all, using PROMs is more popular than using PREMs. The use of patient-reported data is diverse both in terms of the type of disease and the country in which the research is carried out. According to the results of the presented overview, using PROMs and PREMs is most popular in the USA and Netherlands. In the United States, work has been ongoing since 2017 to incorporate additional incentive payments into the Medicare system to achieve desired health goals [98]. In 2004 USA initiated PROMIS (Patient-Reported Outcome Measurement Information System) to improve standards of data collection [99]. The Netherlands is commonly considered a European leader in national registry collection. Some registries include PROMs—for example National Quality Registry for Parkinson’s disease or the low back pain registry. Back in 2007, the country used for the first time value-based payments and introduced a successful bundled system payment for COPD and type-2 diabetes, which included PROMs [8]. Moreover, OECD undertakes numerous initiatives in this subject; for example, it is monitoring the collection of PREMs data in member countries [100]. Based on available data, disease-specific PROMs seem to give the broadest and most specific information on the condition of health of patients (both physiological and psychological) and combine the positive features of generic and state-specific indicators—on the one hand, relative versatility, and on the other, sufficient accuracy [101,102]. According to the presented overview, disease-specific PROMs are most commonly used—especially in the assessment of IC dedicated to COPD and depression. This is due to the availability of recognized research tools such as CAT or SGRQ in COPD or PHQ-9 in depression. PREMs were used less frequently, and the history of their usage is shorter, especially as a condition-specific tool [103] and in specific groups of patients; for example, children. The most significant limitation of the publication is a potential bias resulting from a limited to 10 years period in which eligible articles were published and the inclusion of only full-text articles indexed by PubMed. By deciding on such a research method, it is impossible to determine whether unpublished studies have adopted another form of reporting PROMs and PREMs. However, it seems that the analysis of nearly 7.000 publications allows for an overview of contemporary reporting practice outcomes measures reported by patients. Available studies seem to prove there is a correlation between experience and the effectiveness of procedures [104,105]. Nevertheless, PREMs should be collected and analyzed together with PROMs as some disparities might occur depending on clinical outcomes [7,106]. 

## 5. Conclusions

Using outcomes measures reported by patients remaining under the support of integrated care is varied, both due to the place of research and the type of disease. Interest in these indicators seems to be increasing, especially over the last few years. Nevertheless, it seems necessary to continue work on building research tools for reliable data acquisition, especially in the field of specific PREMs.

## Figures and Tables

**Table 1:** Elements of integrated care.

Elements of IC	N	Percent
Education (incl. education for staff members), self-management, support after discharge	56	76.7
Community-based or home-based care, also a hospital community-based type of care	35	47.9
Support of additional specialists, multispecialty team	35	47.9
Treatment or action plan, treatment coordination, adherence	35	47.9
Others (for example, IT services, home oxygen service, smoking cessation)	33	45.2
Pulmonary rehabilitation	12	52.2 *

* of studies dealing with COPD.

**Table 2:** Total number of assessed outcomes [19,20,21,22,23,24,25,26,27,28,29,30,31,32,33,34,35,36,37,38,39,40,41,42,43,44,45,46,47,48,49,50,51,52,53,54,55,56,57,58,59,60,61,62,63,64,65,66,67,68,69,70,71,72,73,74,75,76,77,78,79,80,81,82,83,84,85,86,87,88,89,90].

Total Number of Instruments Used	N	Percent
<5	21	28.8
6 to 10	26	35.6
11 to 15	13	17.8
15 to 20	2	2.7
>20	5	6.8
n/a	6	8.2
Total	73	100.0

**Table 3:** Main characteristics reporting PROMs and PREMs in evaluation of integrated care.

Author (First)	Year	Country (ISO 3166-1)	Population	Disease	PROMS				PREMS	
					Generic		Disease Specific	Condition or Dimension Specific	Generic	Disease or Condition Specific
					HRQoL	Other than HRQoL				
Afolabi [19]	2013	GBR	199	COPD			CRQ			
							CAT			
Carron [22]	2017	CHE	57	COPD	SF-36		CAT	mMRC		PACIC
							Number of COPD exacerbations reported by patients			
							CRQ			
							SEM-CD			
Henoch [23]	2016	CHE	7810	COPD		Exercise capacity (Likert scale)	CCQ	mMRC		
Davis [24]	2016	CAN	140	COPD		MMAS-8	SGRQ			
Esteban [25]	2016	ESP	119	COPD		HADS	SGRQ	mMRC		
							LCADL			
Gillis [26]	2017	CAN	174	COPD					CTM-3	
									Expectations (single open-ended question)	
									“Helpfulness” of care (Likert scale)	
Garner [27]	2017	GBR	n/a	COPD			CAT		Place of death	
Hernandez [28]	2015	ESP	155	COPD		HADS		mMRC		
						IADL				
Hogg [29]	2012	GBR	1114	COPD		HADS		CRQ-SR		
Jarab [31]	2012	JOR	106	COPD			CSES			
							SGRQ			
Ko [32]	2014	HKG	185	COPD			SGRQ	mMRC		
Ko [33]	2017	HKG	180	COPD			SGRQ	mMRC		
Koolen [34]	2018	NLD	n/a	COPD		PAM	CCQ			CQIAC
						MSQ	NCSI			CSPAM
										PCRS
										CPSET
										PACIC
Kruis [35]	2014	NLD	1086	COPD	EQ-5L	IPAQ	CCQ	mMRC		PACIC
					SF-36	SMAS-30	SGRQ			
Kruis [36]	2010	NLD	1086	COPD	EQ-5L	IPAQ	CCQ	mMRC		PACIC
					SF-36	SMAS-30	SGRQ			
Pinnock [38]	2013	GBR	128	COPD		HADS	SGRQ			
							SECD-6			
							LINQ			
Wu [40]	2015	SGP	62	COPD			CAT			PACIC
Aponte [41]	2017	USA	180	Diab and Obes			DKQ			
Beauregard [43]	2018	CAN	1185	Diab and Obes		Enquête québécoise sur l’activité physique et la santé				
Chwastiak [46]	2017	USA	151	Diab and Obes			PHQ-9			
Ciccone [47]	2010	ITA	1160	Diab and Obes	SF-12					
Fottrell [49]	2016	BGD		Diab and Obes	EQ-5L		SRQ			
Gucciardi [50]	2012	CAN	1200	Diab and Obes					Patients’ experiences and views (in-depth interviews)	
Husted [54]	2014	DNK	71	Diab and Obes		TSRQ-21	PCD		HCCQ	PAID-20
						WHO5				
Jansink [55]	2013	NLD	940	Diab and Obes	VAS scale					
Vermunt [68]	2012	NLD	925	Diab and Obes					Satisfaction about program (Likert scale)	
Zhang [72]	2013	AUS	456	Diab and Obes	SF-12	HADS	DQoL-brief		CSQ-8	
						SMAS-30				
van Eeghen [75]	2018	USA	20	Diab and Obes			PHQ-9		Satisfaction about program (Likert scale)	
Hoffman [77]	2018	USA	97	Diab and Obes	Sizing Me Up	FFQ				
						IPAQ				
						PMI				
						PAQ				
Wake [80]	2012	AUS	120	Diab and Obes	PedsQL	PCSC	BPQ			
						SDQ				
Unützer [82]	2012	USA	7977	Dep			PHQ-9			
Hepner [83]	2011	USA	113	Dep					Satisfaction about program (Likert scale)	
Murphy [84]	2017	VNM	n/a	Dep		WHODAS	SRQ-20			
							CAGE			
Poulsen [85]	2017	DNK	n/a	Dep	EQ-5L	WSAS	BDI-II	PSS	CSQ-8	
					Flanagan QOLS	IPQ	BAI	KES		
						GSS	4DSQ	RTW-SE		
								SPS		
Salisbury [86]	2016	GBR	609	Dep	EQ-5L	HeiQ	PHQ-9	GAD-7	Care coordination (Haggerty)	
						MMAS-8				
						eHEALs				
Sanchez [87]	2017	USA	11895	Dep		PAQ	PHQ-9	GAD-7		
							DKM	SCMHC		
							SD	LSAS		
Von Korff [88]	2011	USA	214	Dep	Quality of life (Likert scale)	WHODAS	SLC-20	SDS		
Wagner [89]	2014	USA	n/a	Dep			PHQ-9			
							MOS-HIV			
Wu [90]	2014	USA	964	Dep	SF-12		PHQ-9	SDS	Satisfaction about program (Likert scale)	

Abbreviations: AUS—Australia; BAI—Beck Anxiety Inventory; BDI-II—Beck Depression Inventory-II; BGD—Bangladesh; BPQ—Body figure perception questionnaire; CAGE—Cut-down, Annoyed, Guilty, Eye-opener Questionnaire; CAN—Canada; CAT—COPD assessment test; CCQ—Clinical COPD Questionnaire; CHE—Switzerland; CPSET—Care Process Self Evaluation Tool; CQIAC—Consumer Quality Index Asthma and COPD; CRQ—Chronic Respiratory Diseases Questionnaire; CRQ-SR—Chronic Respiratory Questionnaire self-report dyspnea scale; CSES—COPD Self-Efficacy Scale; CSPAM—Clinician Support for Patient Activation Measure; CSQ-8—Client Satisfaction Questionnaire; CTM-3—Care Transitions Measure-3; Dep—depression; Diab and Obes—diabetes and obesity; DKM—Depression Knowledge Measure; DKQ—Diabetes Knowledge Questionnaire; DNK—Denmark; DQoL-brief—Diabetes Quality of Life Scale; eHEALs—eHealth Literacy Scale; 4DSQ—Four-Dimensional Symptom Questionnaire; EQ-5L—Euro Qol-5D-5L; ESP—Spain; FFQ—Food Frequency Questionnaire; GAD-7—Generalized anxiety; GBR—United Kingdom; GSS—General Self-Efficacy Scale; HADS—Hospital Anxiety and Depression Scale; HCCQ—Health Care Climate Questionnaire; HeiQ—Health Education Impact Questionnaire; HKG—Hong Kong; IADL—Lawton Instrumental Activities of Daily Living Scale; IPAQ—International Physical Activity Questionnaire; IPQ—Illness Perception Questionnaire; ITA—Italy; JOR—Jordan; KES—Karolinska Exhaustion Scale; LCADL—London Chest Activity of Daily Living; LINQ—Lung information needs questionnaire; LSAS—Latino Scale for Antidepressant Stigma; MMAS-8—Morisky Medication Adherence Scale; mMRC—Medical Research Council scale; MOS-HIV—Medical Outcomes Study HIV Health Survey; MOS-HIV—Medical Outcomes Study HIV Health Survey; MSQ—Marshall Sitting Questionnaire; NCSI—Nijmegen Clinical Screening Instrument; NLD—Netherlands; PACIC—Patient Assessment of Chronic Illness Care Questionnaire; PAID-20- Problem Areas In Diabetes (20 item); PAM—Patient Activation Measure; PAQ—Patient Adherence Questionnaire; PAQ—Patient Adherence Questionnaire; PCD—Perceived Competence in Diabetes Scale; PCRS—Primary Care Recourses and Supports for Chronic Disease Self-Management; PCSC—Harter’s perceived Competence scale for Children; PedsQL—Pediatric Enuresis Module to Assess Quality of Life; PHQ-9—Patient Health Questionnaire-9); PMI—Parent Motivation Inventory; PSS—Perceived Stress Scale; RTW-SE—Return to Work Self-Efficacy; SCMHC—Stigma Concerns about Mental Health Care; SD—Social Distance Scale; SDQ—Strengths and difficulties Questionnaire; SDS—Sheehan Disability Scale; SECD-6—Self-efficacy for managing chronic disease 6 item scale; SEM-CD -Self-Efficacy for Managing Chronic Disease 6-Item Scale; SF-12—Short Form Health Survey; SF-36—Short Form 36; SGP—Singapore; SLC-20—20-item Symptom Checklist Depression Scale; SMAS-30—Self-Management Ability Scale-30; SPS—Stanford Presenteeism Scale); SRQ-20—Self-Reporting Questionnaire; TSRQ -21—21-item Treatment Self-Regulation Questionnaire; USA—United States of America; WHO5—World Health Organization-5 scale; WHODAS—World Health Organization Disability Assessment; WSAS—Work and Social Adjustment Scale.
